# Transplant of microbiota from long-living people to mice reduces aging-related indices and transfers beneficial bacteria

**DOI:** 10.18632/aging.102872

**Published:** 2020-03-16

**Authors:** Yinfeng Chen, Siyuan Zhang, Bo Zeng, Jiangchao Zhao, Mingyao Yang, Mingwang Zhang, Yan Li, Qingyong Ni, De Wu, Ying Li

**Affiliations:** 1 Farm Animal Genetic Resources Exploration and Innovation Key Laboratory of Sichuan Province, Sichuan Agricultural University, Chengdu, Sichuan, China; 2 Department of Animal Science, Division of Agriculture, University of Arkansas, Fayetteville, AR 72701, USA; 3 Institute of Animal Nutrition, Sichuan Agricultural University, Chengdu, Sichuan, China

**Keywords:** longevity, gut microbiota, fecal microbiota transplantation, aging-related index, healthy aging

## Abstract

A close relationship between age and gut microbiota exists in invertebrates and vertebrates, including humans. Long-living people are a model for studying healthy aging; they also have a distinctive microbiota structure. The relationship between the microbiota of long-living people and aging phenotype remains largely unknown. Herein, the feces of long-living people were transplanted into mice, which were then examined for aging-related indices and beneficial bacteria. Mice transplanted with fecal matter from long-living people (L group) had greater α diversity, more probiotic genera (*Lactobacillus* and *Bifidobacterium*), and short-chain fatty acid producing genera (*Roseburia, Faecalibacterium, Ruminococcus, Coprococcus*) than the control group. L group mice also accumulated less lipofuscin and β-galactosidase and had longer intestinal villi. This study indicates the effects that the gut microbiota from long-living people have on healthy aging.

## INTRODUCTION

The interactions between gut microbiota and their host(s) have become a popular topic in research. There is growing evidence to suggest that a close relationship exists between gut microbiota and aging [1, 2]. Age-related changes in gut microbiota occur widely among animals, with evidence of this ranging from insects to mammals [3, 4]. Human-based studies have revealed a trend in age-related microbiota features, which shows an increase in gut microbiota diversity from infants to adults, followed by a decrease as adults age [5]. Biagi et al. [6] found signatures of extreme longevity in gut microbiota composition that were related to extreme aging. Combined with the data from Biagi et al. Kong et al. found 11 features shared among long-living Chinese and Italian people, including higher alpha diversity and operational taxonomic units (OTUs) [7]; they also showed that long-living people had greater gut microbiota diversity than a younger group among Chinese and Japanese populations [8].

High microbiota diversity has been associated with good health in general [9]. Early research on the gut microbiota of elderly people has indicated that healthier subjects have significantly greater gut microbiota diversity than those in long-term residential care [10]. Overall, the information obtained from studies such as these suggests that long-living people can serve as an acceptable model to investigate whether gut microbiota is a feasible target for promoting healthy aging. However, the exact roles that the microbiota play still require investigation.

Studies in animal subjects have shown that age-related microbiota can affect the lifespan of the host [11]. Ten-day-old and 30-day-old *Drosophila* were used as microbiota donors for 10-day-old *Drosophila*. The lifespan of the 10-day-old transplant group lived significantly longer than the 30-day-old transplant group, and had a decreased frequency in intestinal barrier dysfunction. Subsequently, Smith et al [12] transplanted the gut contents of young and old African turquoise killifish to old fish. Consistent with the results from *Drosophila*, fish transplanted with feces from young donors had a longer lifespan and were significantly more active. These results suggest that the gut microbiota of young individuals can slow host aging and prolong the lifespan of the tested species.

**Figure 1:**
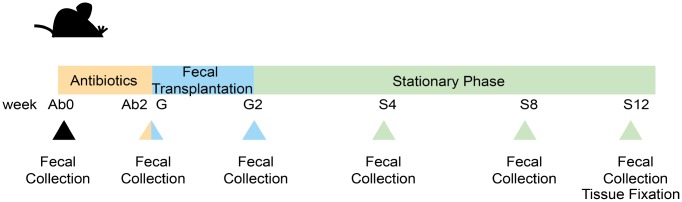
**Experimental design and samples collection.** The whole experiment was divided into 3 phases, totaling 7 sampling points set at the following: before Ab treatment (Ab0); after Ab treatment(Ab2); the first day at FT (G); the last day at FT (G2); 4 weeks after FT (S4); 8 weeks after FT (S8); 12 weeks after FT (S12).

In economically developed countries long-living people (>90 years old) account for approximately 1/5000-1/10000 of the population and usually present good health and mental outlooks, have lower hospitalization rates, and shorter hospitalization times than the general population over their lifetime [13–15]. They also present a delayed onset or absence of senile diseases, such as cardiovascular disease, Alzheimer's disease, and cancer [14, 16, 17]. Previous studies indicate that cancer prevalence in the age groups 60–80 years old range between 25% to 42%, but cancer incidence and cause of death presented a threefold decrease after 90 years old and reached 0–4% above 100 [18]. Therefore, long-living people have been regarded as a suitable model for healthy aging [15]. In the current study, the hypothesis that the gut microbiota of long-living people has the ability to delay host aging compared with those of average lifespan, is tested. To test this hypothesis, the gut microbiota of long-living and typical aging elderly people were transplanted into antibiotic-treated mice, which were then analyzed for differences in gut microbiota and aging indices (Figure 1). L group mice demonstrated greater microbiota diversity and beneficial bacteria, such as probiotic genera and short-chain fatty acid producers. Importantly, aging-related indices, such as lipofuscin and β-galactosidase accumulation, were less in the L group. Our experiment provided primary evidence that the gut microbiota of long-living people has the ability to delay host aging.

## RESULTS

### Aging-related index assessment

**Figure 2:**
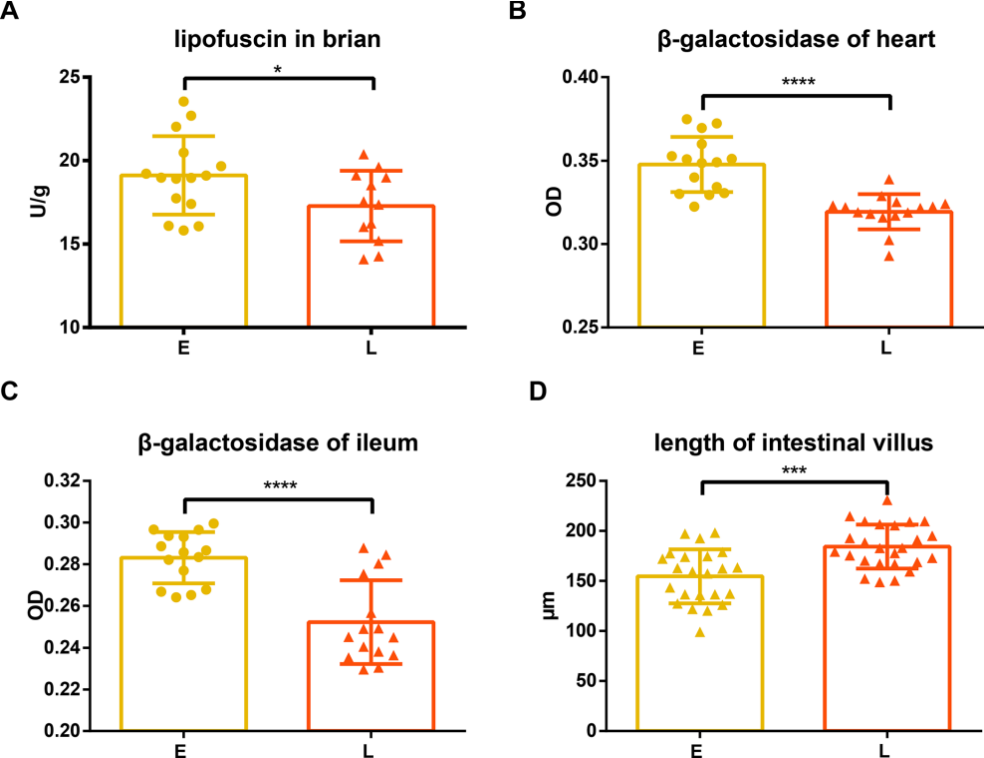
**Difference in aging related indices between groups E and L.** (**A**) and (**D**) Lipofuscin in brain and length of intestinal villus are shown on. The level of β-gal in both the (**B**) heart and (**C**) ileum in E group and L group. *p<0.05, unpaired t test, ***p<0.0001, ****p<0.001, Mann-Whitney U test.

Lipofuscin and β-galactosidase were measured in different tissues of the two groups. Compared to the E group mice, L group mice had significantly lower lipofuscin in the brain tissue (Figure 2A, *p*<0.05). Similarly, the levels of β -galactosidase in the heart and ileum tissue of L group mice were significantly less than those in the E group mice (Figure 2B and 2C, *p*<0.0001).

Superoxide dismutase (SOD), glutathione peroxidase (GSH-PX), and malondialdehyde (MDA) in serum were also measured. SOD and GXH-PX activities were not significantly different between L group and E group mice (Supplementary Figure 1A and 1C; *p*=0.2386; 0.2597, respectively), although activity values from the L group mice tended to be greater. Consistent with this observation, there was no significant difference in MDA levels between the E group and L group mice (Supplementary Figure 1B, *p*=0.1334).

The intestinal villi of the L group mice were significantly longer than that of the E group mice (Figure 2D, *p*<0.001), while there was no significant difference in crypt depth between L group and E group mice (Supplementary Figure 1D, *p*=0.1542).

### 16S rRNA sequence analysis

**Figure 3:**
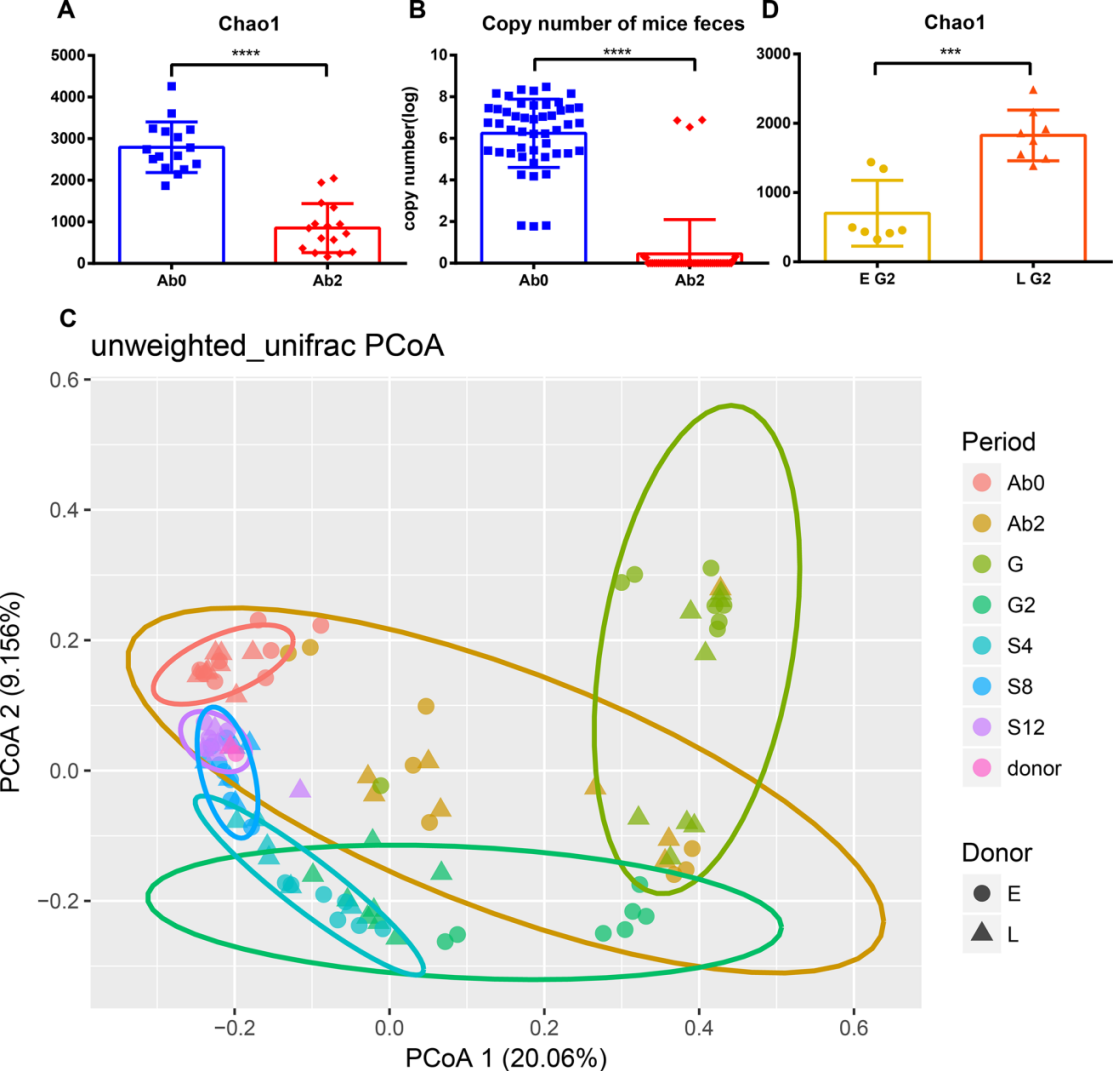
**Efficient antibiotic treatment and transplant.** Antibiotic treatment reduces (**A**) Chao1 diversity and (**B**) bacterial copy number. (**C**) Principal Coordinate Analysis of mice and donor gut microbiomes based on unweighted UniFrac distance. Triangles represent the L group and circles represent the E group. Different colors represent different time periods. (**D**) The Chao1 diversity of E group and L group after transplantation. ***p <0.001, ****p<0.0001, Mann-Whitney U test.

The change in gut microbiota was analyzed after the transplantation process. Ninety-nine mice fecal samples and two fecal suspensions from long-living and elder donors were collected for 16S rRNA sequencing and analysis. In total, 6,118,012 high-quality reads corresponding to 22,745 OTUs after filtering for chimeras and low-quality OTUs were identified, which were annotated to 753 genera. Three alpha diversity metrics were calculated to assess the diversity of donors and mice during the whole experiment (Supplementary Figure 2), including Shannon index, Chao1 index, and observed species. As expected, antibiotic treatment significantly decreased the diversity and total bacterial copy number in the mice (Figure 3A, 3B, and Supplementary Figure 3A, 3B, *p* <0.0001). Unweighted UniFrac distances were used to assess the relationship between the community structure of donors and mice, which was visualized using Principal Coordinates Analysis (PCoA) (Figure 3C). As the number of days increased, donors and recipients clustered together at S12. Alpha diversity increased slightly after the FT (fecal transplantation) phase (G2) and the increasing trend was gradual during the stationary phase (from S4 to S8). Interestingly, the rate of increase in Chao1 diversity was different between the two groups during the FT phase (from G to G2) (Supplementary Figure 2A and 2B). The Chao1 diversity of the L group at G2 was significantly greater than at G (Supplementary Figure 2B, *p*<0.05). However, there was no significant difference in Chao1 diversity in the E group between G and G2 phase (Supplementary Figure 2A). Consistent with this result, the alpha diversity of the L group at G2 was significantly greater than that of the E group (Figure 3D and Supplementary Figure 3C, 3D, Chao 1, observed species, *p*<0.001; Shannon, *p*<0.05).

### Bacterial genera differences between transplant recipients

**Figure 4:**
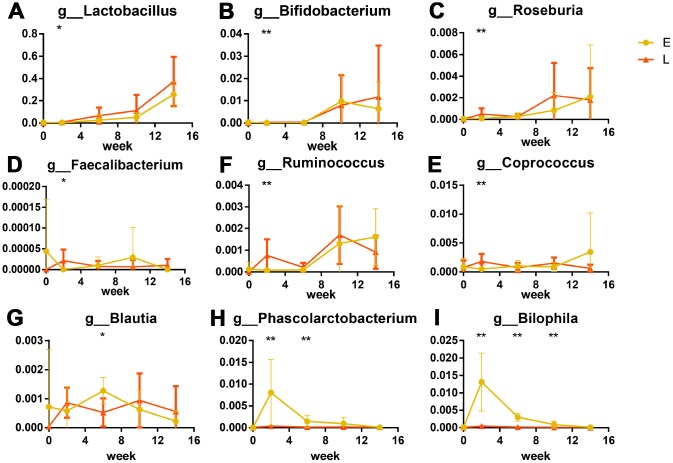
**Significant differences in bacteria in different groups after transplantation (%): Probiotics were significantly greater in abundance in the L group than in E group.** (**A**) *Lactobacillus*, (**B**) *Bifidobacterium*. Short chain fatty acid producers were greater in abundance in the L group than in E group are shown as box plots: (**C**) *Roseburia*, (**D**) *Faecalibacterium*, (**E**) *Ruminococcus*, (**F**) *Coprococcus*. (**G**) *Phascolarctobacterium* and (**H**) *Blautia* were more abundant in the E group. The opportunistic pathogen significant higher in E group: *Bilophila wadsworthia* (**I**) *p<0.05, **p<0.01 by LEfSe analysis.

LEfSe analysis was used at the genus level to identify the differences in bacterial genera between the E and L groups. Eighty-seven genera were differential in total (Supplementary Figure 4, LDA cut-off = 2, *p*<0.05). A peak was observed in the number of significantly differential genera in the L group at G2. Forty-two genera were significantly more abundant in the L group than in the E group (Supplementary Figure 4). The relative abundance of *Lactobacillus* at G2 was significantly greater in the L group compared to the E group (Figure 4A, *p*<0.05); this same trend was observed throughout the entire experiment. The abundance of *Bifidobacterium* was significantly greater in the L group compared to the E group at G2 (Figure 4B, *p*<0.01).

Six genera that produce short-chain fatty acids (SCFAs) were significantly different between the L and E groups. Of these, four genera (*Roseburia, Faecalibacterium, Ruminococcus, and Coprococcus*) at G2 were more abundant in the L group than that in the E group (Figure 4C–4F, *p*<0.05). Two genera (*Blautia* and *Phascolarctobacterium*) were at S4 more abundant in the E group than that in the L group. (Figure 4G, 4H, *p*<0.05). Notably, *Bilophila* were significantly abundant in the E group from G2 to S8 than that in the L group (Figure 4I, *p*<0.01) both at the genus level and OTU level. Cross-referencing data with the NCBI BLAST database led to the annotation of the *Bilophila* species as *Bilophila wadsworthia*.

## DISCUSSION

Thevaranjan et al. demonstrated that age-related alteration of the microbiota can drive age-associated inflammation and intestinal permeability [19]. The intestinal permeability of young germ-free mice was greater when transplanted with the gut microbiota of old mice than when transplanted with that of young mice. Another experiment obtained a similar result in terms of systemic inflammation [20]. Interestingly, Cui et al. found that fecal microbiota transplantation improved the survival rate of irradiated mice, but microbiota transplantation from older mice failed to improve the survival rate [21]. Together, this evidence suggests that some beneficial component of gut microbiota may be lost with age, or some harmful effects may accumulate. Gut microbiota transplanted from younger donors in Drosophila melanogaster and African turquoise killifish have longer lifespans than those who have received transplants from old donors [11, 12]. Studies have shown that there are signatures of extreme longevity in the human gut microbiota, which differ from that of standard aging individuals [2, 6–8]. Therefore, we hypothesize that the gut microbiota from long-living people can slow down aging, which was tested using microbiota transplantation.

Lipofuscin (age pigments) and senescence-associated β-galactosidase have been widely used as biomarkers for aging and replicative senescence, respectively [22–25]. Lipofuscin is an autofluorescent, non-degradable pigment associated with age, which accumulates because of phagocytosis and autophagocytosis of modified cellular materials within secondary lysosomes of postmitotic cells [22, 26]. The amount of lipofuscin accumulated in neuronal cells increases with age [27, 28] and positively correlates with the production of reactive oxygen species (ROS) and mitochondrial damage [29, 30]. Our results show that lipofuscin accumulated in the brain tissue of L group mice significantly less than in that of E group mice (Figure 2A), suggesting E group mice may have had an increased incidence of oxidative stress, and damaged, defective, impaired, and giant mitochondria with a low rate of degradation [29–33].

Dimri et al. first proposed that β-galactosidase activity that is detectable at pH 6.0 can be defined as senescence-associated β-galactosidase (SA-βgal) activity [24]. This has become a well-known biological marker for replicative senescence [34–36]. Experiments have shown that the percentage of SA-βgal-positive cells was significantly greater in the heart tissue of old mice than that of young mice [37, 38]. However, Kurz et al. demonstrated that SA-βgal activity is a sign of residual lysosomal activity at a suboptimal pH, which is detectable due to the increased lysosomal content in senescent cells [39]; they also showed that an increase in SA-βgal activity was closely linked to an increase in β-galactosidase protein levels. Another study confirmed that increased SA-βgal activity in senescent cells is due at least in part to increased levels of β-galactosidase [40]. Our results showed that the tissues of L group mice accumulated less β-galactosidase than E group mice (Figure 2B and 2C), suggesting that senescent cells accumulated in these tissues had a decreased rate of senescent cells generation or an increased rate of senescent cells clearance in L group mice. In addition, it has been reported that the accumulation of lipofuscin and SA-βgal are both associated with lysosomal dysfunction caused by cellular senescence [26, 33, 39, 40]. Therefore, it is likely that fecal microbiota transplantation from long-living people might reduce age-related lysosomal dysfunctions of the recipients because of the reduced lipofuscin and β-galactosidase in the L group mice.

Both SOD and GSH-PX are important antioxidant enzymes to scavenge oxygen free radicals, which cause oxidative damage of cells and correlate with the rate of aging in animals [41–43]. MDA is a product of lipid peroxidation, which reflects the level of cellular oxidative damage [44]. All three indices have been proposed as markers of aging [42]. The SOD and GSH-PX activities were numerically greater whereas the MDA was lower in the L than in the E group mice, suggesting that fecal microbiota transplantation from the long-living people decreased aging related oxidative damage measures (Supplementary Figure 1A–1C). It is worth noting that these measures were only numerically different, but not statistically significant. Future studies with a larger sample size, greater fecal microbiota dosage, and/or longer transplantation duration are desired to reach a statistical significance.

Previous work has indicated that the intestinal capillaries of germ-free mice develop poorly compared to conventional mice, suggesting that the microbiota contributes to the development of intestinal villi [45]. Moreover, the ilea of aged mice were found to exhibit distinct histological features, characterized by a reduction in villus length [46]. We found that the L group mice had longer intestinal villi than those observed in the E group mice (Figure 2D), indicating that the L group has a higher absorptive capacity and younger histological features than the E group.

Together, results from the discussed physiological indices suggest that the gut microbiota from long-living people could carry an anti-aging function. 16s rRNA results from transplanted mice indicate that the L group mice had more beneficial bacteria (Figure 4). Numerous studies have shown that *Lactobacillus* and *Bifidobacterium* have beneficial effects on diseases such as inflammatory bowel disease, obesity, and type 2 diabetes mellitus [47–50]. Remarkably, *Lactobacillus* and *Bifidobacterium* have been linked to a prolonged lifespan in *Caenorhabditis elegans*, and were found to reduce oxidative stress and lipofuscin accumulation [51, 52]. Exopolysaccharides produced by *Bifidobacterium*, which was isolated from the feces of Chinese centenarians, inhibited lipid peroxidation and reduced lipofuscin accumulation in mouse brain tissue [53]. Overall, the results from these studies are in agreement with our lipofuscin results, and support the hypothesis that the gut microbiota of long-living people can delay host aging more than that of a typical person.

Further analyses used a Linear discriminant analysis (LDA) coupled with effect size (LEfSe) [54] revealed that six SCFA (such as acetate, propionate, and butyrate) producers, which belong to nine predominant genera co-occurred in Chinese healthy young adults [55], enriched in L group mice (*Roseburia, Faecalibacterium, Ruminococcus and Coprococcus*) (Figures 4C–4F) and E group mice (*Blautia, Phascolarctobacterium*) (Figures 4G, 4H). SCFAs, especially butyrate, decrease the apoptosis of epithelial cells, and increase the length of intestinal villus in both mice and weaned piglets [56–59]. Butyrate also improves the functions of the intestinal barrier [58, 59], which are related to inflammation [19]. Interestingly, intestinal barrier dysfunction was previously associated with systemic inflammation that shortened the lifespan of *Drosophila melanogaster* [11, 60]. Taken together these results suggest that enriched SCFA producers in the L group mice might contribute to longer intestinal villi and less β-galactosidase. Especially, four SCFAs producers enriched in the L group mice were butyrate-producing bacteria [61–65]. Butyrate was previously linked to increased adhesion of *Lactobacillus* and *Bifidobacterium to* intestine [66], which coincides with the greater abundance of probiotic species in the L group mice mentioned above.

Our LEfSe analysis found that *Bilophila wadsworthia*, which is considered to be an opportunistic pathogen [67] that causes systemic inflammatory response [68, 69], were enriched in the E group mice (Figure 4I). Probiotic species and SCFA producers were previously found to inhibit infection by opportunistic pathogens by niche occupation [66, 70, 71], which could explain why less *Bilophila wadsworthia* were observed in the L group mice.

### Study limitations

Firstly, this study has focused on the effects of gut microbiota transplantation from elder and long-living humans to mice; therefore, further studies using a younger transplant group would be useful for understanding the relationship between age-related gut microbiota and aging. Secondly, a small sample size of 1 donor and 10 recipients per group was used, which means results may be difficult to generalize to a wider population. Huge inter-individual variation has been reported for the human gut microbiota [5, 72]. Hence, our future studies will include larger sample sizes and younger subjects to further explore the role age-related gut microbiota play in aging.

## CONCLUSIONS

In conclusion, results from this study indicate that mice transplanted with gut microbiota from long-living people have more beneficial bacteria and lower metabolites related to aging. This demonstrates the potential use of gut microbiota from long-living people for anti-aging purposes and to promote healthy aging. We also believe that the relationship between aging and gut microbiota is complex. Findings from this study support the hypothesis that age-related microbiota can affect the aging of hosts long-term. Our results provide new insight into healthy aging. Further studies are needed regarding the microbiota’s role in long-living people, and also for the role the gut microbiota plays in aging.

## MATERIALS AND METHODS

### Animal study design and treatments

Eleven-month-old male C57BL/6 mice (n=16) were obtained from Chengdu Dossy Experimental Animal Co. Ltd., which were housed in a single cage in a specific pathogen free level animal facility at the Animal Nutrition Institute of Sichuan Agricultural University. All mice were kept under 12/12 hours light/dark cycle and 25 °C and were fed irradiated food and autoclaved water *ad libitum.*

For antibiotic (Ab) treatment, mice were treated for 2 weeks with 1 g/L ampicillin neomycin trisulfate salt hydrate, and metronidazole, and 0.5 g/L vancomycin (Sangon Biotech, Shanghai, China) [73] in their drinking water. After Ab treatment, mice were randomly divided into two groups according to their weight. Fecal matter from the long-living person was transplanted into L group mice and that from the elderly person was transplanted to E group mice. Mice were given 0.2 mL fecal suspension by gavage once a day for 2 weeks. Both volunteers were male, from Dujiangyan, Sichuan province, China, who were both in good health. The long-living person was 101 years old and the elderly person was 70 years old. Human participant ages were divided into two stages according to Odamaki and Biagi’s work [5, 6]. Neither had a history of gastrointestinal diseases or record of antibiotic used within 3 months. Feces were collected at the volunteers’ homes and put into a sterile fecal collection tube, then transferred to the laboratory within 24 hours in an ice box, and stored at -80 °C immediately. The method of making fecal suspension was slightly adjusted with Turnbaugh’s [74]. In brief, suspensions were prepared by diluting 1 g frozen human fecal sample in 10 mL PBS containing 15% glycerol (v/v), under anaerobic conditions. The mixed material was then suspended by vortex, and stored at -80 °C after split charging.

The experimental design, including the time points of sample collection, is shown in Figure 1. Fresh fecal samples were immediately transferred into liquid nitrogen container before they were stored at -80 °C.

### Beta-galactosidase assay

At S12, all mice were anesthetized and killed by cervical dislocation. Heart and ileum tissues were collected for histology by fixing in 4% paraformaldehyde solution overnight. Five-micrometer paraffin sections were processed for immunohistochemistry (IHC) staining and periodic acid Schiff reaction (PAS) staining. For IHC, a primary antibody against beta-galactosidase (Bioss, Beijing, China) was used. The expression level of beta-galactosidase in each tissue was determined by integrated optical density (IOD) and area. The average density (MD) of each microscopic field (objective 400×) was calculated. Five fields PAS stained paraffin sections of ileum tissue were randomly selected and the length of intestinal villi and crypt depths were measured.

### Lipofuscin assay

Lipofuscin concentrations were determined by the lipofuscin extraction method [75]. In short, single mouse brains were homogenized in chloroform-methanol solution (2:1, v/v) to make a 1:20 suspension. An equal volume of distilled water was added, then the solution was mixed using a vortex. After centrifugation at 3000 rpm for 10 min, lipofuscin levels were analyzed from the chloroform phase using a Varioskan LUX Multimode Microplate Reader (Thermo Scientific, USA) based on excitation (360 nm) and emission (420 nm) maxima. Using the fluorescence intensity of quinine sulphate (1 μg/ml) as a unit, the concentration of lipofuscin was expressed in units per g tissue wet weight.

### DNA extraction and bioinformatics

Total bacterial DNA was extracted from fecal samples by using a Magnetic Soil and Stool DNA Kit (TIANGEN Biotech, Beijing, China) according to manufacturer’ s instruction, and was stored at -80 °C before further analysis. Sequencing was performed at the Novogene Bioinformatics Technology Co., Ltd. Briefly, V3-V4 of the 16S rRNA gene was amplified using the 341F/806R barcode primer pair. All PCR reactions were carried out in 30 μL reactions with 15 μL of Phusion® High-Fidelity PCR Master Mix (New England Biolabs); 0.2 μM of forward and reverse primers, and about 10 ng template DNA. Thermal cycling consisted of initial denaturation at 98 °C for 1 min, followed by 30 cycles of denaturation at 98 °C for 10 s, annealing at 50 °C for 30 s, and elongation at 72 °C for 60 s, with a final 72 °C for 5 min. PCR products were purified using a GeneJET Gel Extraction Kit (Thermo Scientific, USA). Sequencing libraries were generated using a NEB Next® Ultra™ DNA Library Prep Kit for Illumina (NEB, USA) following the manufacturer’s recommendations. Amplified libraries were sequenced on an Illumina MiSeq 2 × 250 platform by Novogene (Beijing, China).

Sample reads were assembled by using Qiime software [76]. Chimeric sequences were removed using USEARCH v8.0 [77], and operational taxonomic units (OTUs) were picked de novo with a 97% similarity threshold. Taxonomy assignment of OTUs was performed by comparing sequences to the SILVA database (http://www.mothur.org/wiki/Silva_reference_ files). Alpha diversity analysis included Shannon index, Chao1, and observed species. Jackknifed beta diversity was represented by unweighted Unifrac distances, calculated with 10 times of subsampling, and these distances were visualized by Principal Coordinate Analysis (PCoA) using R v3.4.3.

### Bacterial quantification by real-time PCR

qPCR analysis of bacterial 16S rRNA copy number was used to quantify the effect of antibiotic treatment. The forward and reverse primers were used as follows: 341F 5′-CCTACGGGAGGCAGCAG-3′; 519R 5′-ATTACCGCGGCTGCTGG-3′ [78]. The 10 μL reaction mixture contained 5 μL SYBR® Premix Ex Taq™ II (Takara, Beijing, China),1 μL DNA (samples or diluted plasmid standards), 0.4 μL (10 μM) of each primer, 3.2 μL ddH_2_O. The PCR program consisted of a DNA denaturation step at 95 °C for 30 s, 40 cycles with an annealing step at 95 °C for 5 s and an elongation step at 60 °C for 30 s. To determine the specificity of amplification, a melting curve was created after the end of amplification.

Standard curves were constructed by PCR product of the 16S rRNA gene of *E.coli*. *E.coli* DH5α genomic DNA was extracted from bacterial liquid using a TIANamp Bacteria DNA Kit (TIANGEN Biotech, Beijing, China). Primers and reaction mixture were used as above, except that SYBR Premix was replaced with Premix Taq™ (Takara, Beijing, China). The PCR program consisted of pre-degeneration at 94 °C for 3 min, 30 cycles with denaturation at 94 °C for 30 s, annealing at 60.6 °C for 30 s, elongation at 72 °C for 30 s and final elongation step at 72 °C for 5 min. PCR products were purified using a Universal DNA Purification Kit (TIANGEN Biotech, Beijing, China). DNA inserts were linked with the pMD19-T vector (Takara, Beijing, China), transformed, plated, and single colonies were selected. Colonies were cultured and plasmids were extracted according to manufacturer’s instruction (Takara, Beijing, China) [79]. Plasmids were sequenced to verify successful clones. The concentration of plasmid standard was measured using a spectrophotometer (Thermo Scientific, USA). The target sequence copy number was calculated as previously described [80]. Gradient dilutions were performed to provide a template for the standard curve. The function between threshold cycle (C_t_) and log copy number (x) was C_t_ = -0.3172 * x + 12.289, R= 0.9981.

### Statistical analyses

Before significance testing, all data were checked by normal distribution. An Ordinary one-way ANOVA or unpaired t-test were used for significance tests if conforming to a normal distribution, otherwise, a Kruskal-Wallis test or Mann-Whitney test was used. Dunn's multiple comparisons test was used for multiple comparisons. Linear discriminant analysis (LDA) coupled with effect size (LEfSe) [54] was used to identify abundance differential genera or OTUs between groups. GraphPad 6.0 was used for significance testing and visualization. R packages “bindr”, ”dplyr”, ”ggpubr”, ”ggplot2”, ”vegan”, and “reshape2” were used for plotting.

### Ethics approval

This study was approved by the Institutional Animal Care and Use Committee of the Sichuan Agricultural University under the permit number DKY-S20160911. All experiments were performed in accordance with the approved guidelines and regulations.

## Supplementary Material

Supplementary Figures
