# Supplementary material for: Transplant of microbiota from long-living people to mice reduces aging-related indices and transfers beneficial bacteria
Source: Aging (Albany NY). 2020 Mar 16;12(6):4778–93. doi: 10.18632/aging.102872 (PMC7138539; doi:10.18632/aging.102872)
Supplement: Supplementary Figures [file aging-12-102872-s001..pdf]

SUPPLEMENTARY FIGURES

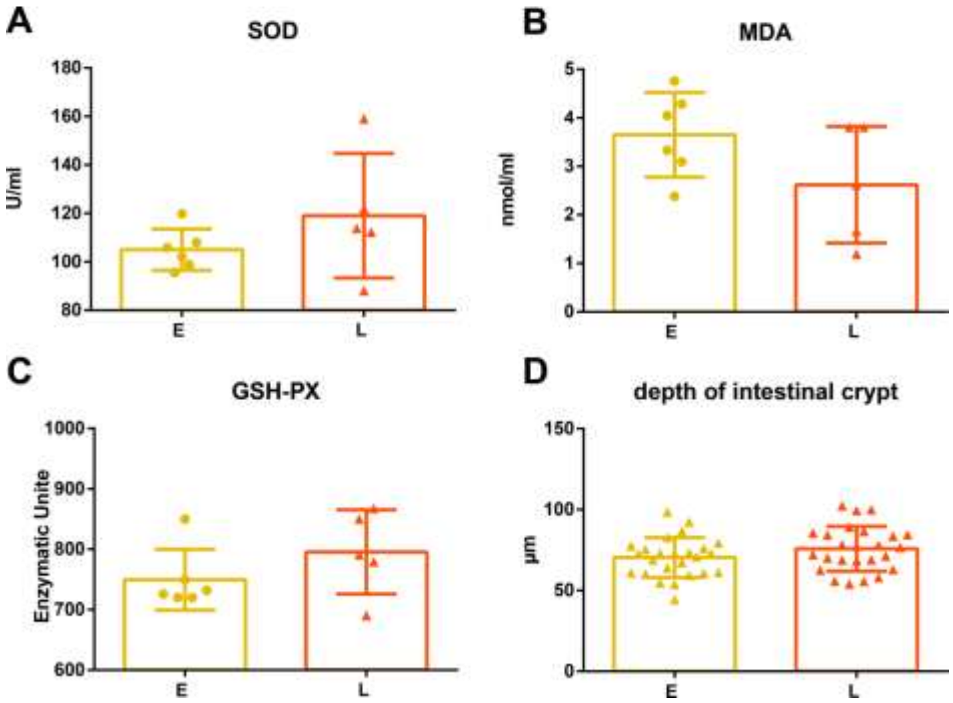

Supplementary Figure 1. Antioxidant level in serum and depth of intestinal crypts. (**A**) Superoxide dismutase (SOD), (**B**) malondialdehyde (MDA), (**C**) glutathione peroxidase (GSH-PX), (**D**) depth of intestinal crypt. Unpaired t test.

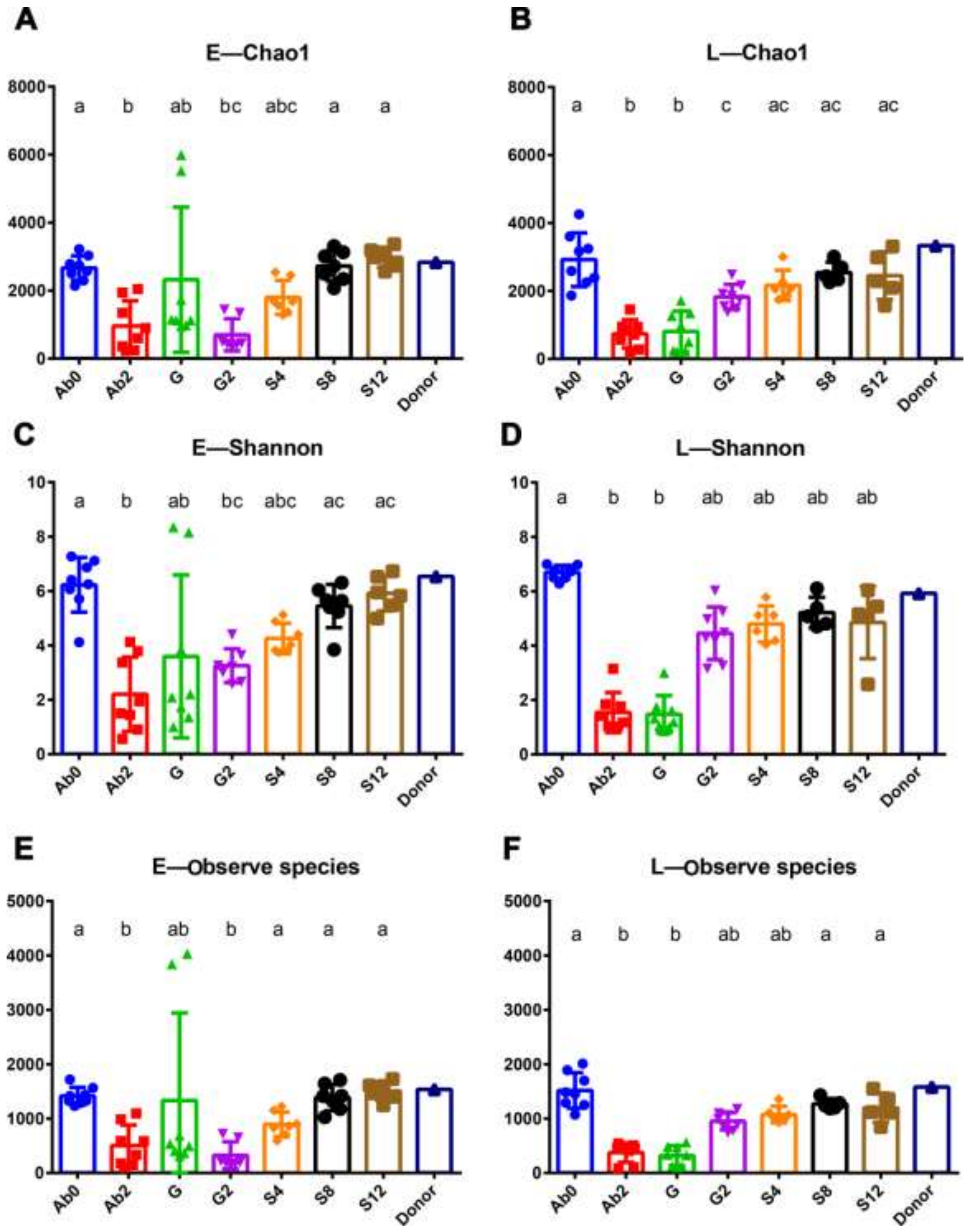

Supplementary Figure 2. Alpha diversity in each period between groups. Three metrics are shown, including (**A**, **B**) Chao1 index, (**C**, **D**) Shannon index, and (**E**, **F**) Observed species in both mice and donors. Dunn's multiple comparison test was used for multiple comparisons. Letters indicate inter-group significance. Lowercase letters represent *p*<0.05.

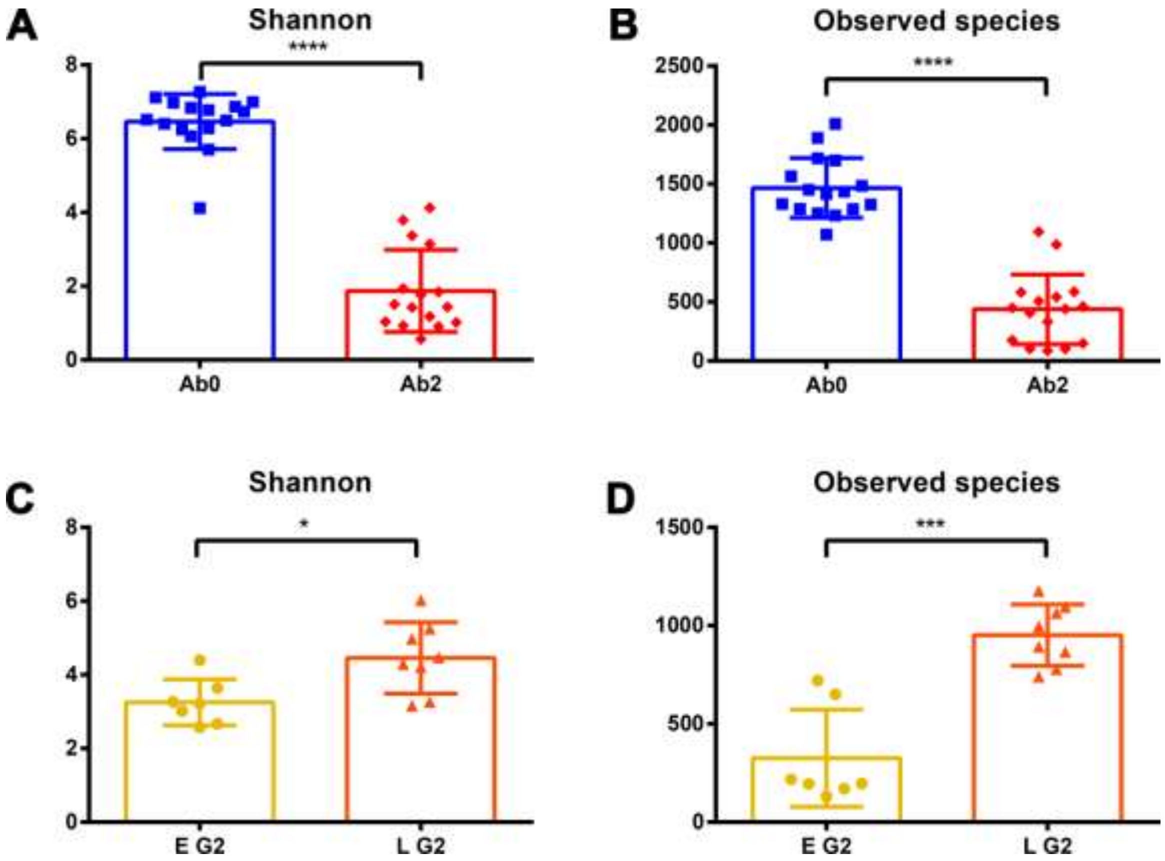

Supplementary Figure 3. Comparison of the alpha diversity of the gut microbiome after antibiotic treatment. (**A**, **C**) Shannon diversity index. (**B**, **D**) number of observed OTUs. **p*<0.05, ****p*<0.001, *****p*<0.0001, Mann-Whitney U test.

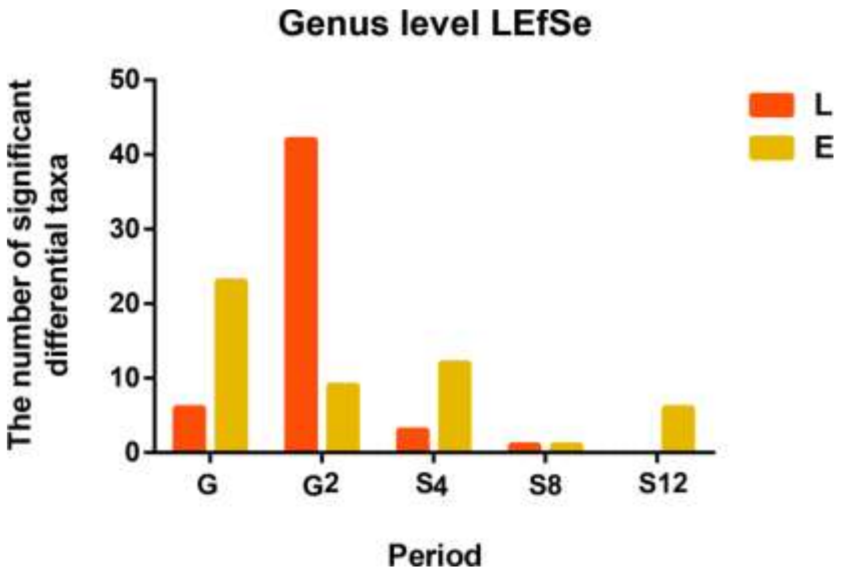

Supplementary Figure 4. The number of significant differential taxa determined by LEfSe analysis at the genus level and OTU level. LDA cut off=2, *p*<0.05.
